# Gut permeability and low-grade inflammation in bipolar disorder

**DOI:** 10.1192/j.eurpsy.2023.835

**Published:** 2023-07-19

**Authors:** M. Couce, G. Paniagua, L. González-Blanco, A. García-Fernández, C. Martínez-Cao, P. Sáiz, J. Bobes, M. P. García-Portilla

**Affiliations:** 1 Central University Hospital of Asturias. Health Service of the Principality of Asturias; 2 Department of Psychiatry, University of Oviedo; 3 Health Institute Research of the Principality of Asturias (ISPA); 4 Biomedical Research Networking Centre in Mental Health (CIBERSAM); 5 Institute of Neurosciences of the Principality of Asturias (INEUROPA), Oviedo, Spain

## Abstract

**Introduction:**

Systemic inflammation has been increasingly related to bipolar disorder -BD- (Tanaka et al. Neurosci Res 2017;115 59-63). Intestinal bacterial translocation has been postulated as one of the causes of this inflammation (Nguyen et al. J Psychiatr Res 2018;99 50-61). A possible pathway is through the lipopolysaccharide, which is presented to CD14 through lipopolysaccharide binding protein (LBP) leading to a release of systemic inflammatory markers like C-reactive protein (CPR) (Funda et al. Infect Immun 2001;69 3772-81).

**Objectives:**

1) Describe gut permeability in patients with BD through the determination of intestinal inflammatory markers (LBP, sCD14) in plasma; 2) Analyze variables associated with intestinal inflammation.

**Methods:**

Cross-sectional study of 38 patients with BD [mean age=45.50 (SD=10.93; range 23-68); males=15 (39.5%)], recruited from mental health outpatient clinics in Oviedo (Spain).

Assessment: Pro-inflammation biomarkers [CRP (mg/dL), Erythrocyte Sedimentation Rate (ESR) (mm/h), Neutrophil/Lymphocyte, Monocyte/Lymphocyte, Platelet/Lymphocyte and Systemic Immune Inflammation Indexes]. Indirect markers of intestinal bacterial translocation [LBP, soluble CD14 (sCD14)]. Dichotomous variables were created for LBP, considering LBP ≥15 μg/dL as increased gut permeability; and for CPR, considering CRP≥0.3 as systemic inflammation. Metabolic syndrome [ATPIII criteria: glucose, HDL, triglycerides (mg/dl), arterial pressure (mmHg), abdominal circumference (cm)], body mass index (BMI) (kg/m2), smoking, cannabis or alcohol use.

Statistical analyses: t-Student test, multiple linear regression analyses.

**Results:**

Average LBP was 14.60 μg/dL (SD=6.4) and 15 patients (39.5%) had increased gut permeability. Moreover, average CPR was 0.40 mg/dL (SD=0.58) and 16 patients (47.1%) showed systemic inflammation. There were no patients with increased levels of sCD14.

Associations were found between LBP and CPR (r=0.357; p=0.032), cannabis use in the last month (t=-2.293; p=0.029), BMI (r=0.433; p=0.008) and abdominal obesity (t=3.006; p=0.005); but no with age or sex.

Subsequently, a multiple linear regression model for LBP was calculated with variables previously mentioned, and age (based on expert criteria). The overall regression was statistically significant (R2=0.49, F=9.273, p<0.001). It was found that CPR, abdominal obesity, and cannabis use in the last month significantly predicted LBP levels (table 1).

**Table 1.** Multiple linear regression analyses to LBP

	B	SE	β	t	p
CPR	4.842	1.529	0.439	3.167	0.004
Abdominal obesity	4.810	1.849	0.362	2.601	0.014
Cannabis use	-5.048	2.273	-0.296	-2.221	0.034

**Conclusions:**

More than one third of patients with BD had increased gut permeability. Almost 50% had systemic inflammation. Intestinal permeability was directly related to abdominal obesity and systemic inflammation, but inversely related to cannabis use.

**Disclosure of Interest:**

None Declared

